# Impact of Task Trainer‐Based Training on Medical Students' Technical Skills, Nontechnical Skills, and Confidence in Peripheral Intravenous Cannulation: A Single‐Group Pre–Post Study

**DOI:** 10.1002/hsr2.73307

**Published:** 2026-10-05

**Authors:** Maher Abdessater, Ramy Touma Sawaya, Sandra Elias, Joanne El Turk, Pascal Roquet, Raghid El Khoury

**Affiliations:** ^1^ Division of Urology, Department of Surgery American University of Beirut Medical Center Beirut Lebanon; ^2^ Doctoral College Holy Spirit University of Kaslik (USEK) Jounieh Lebanon; ^3^ Department of Anesthesiology, Gilbert and Rose‐Marie Chagoury School of Medicine Lebanese American University Byblos Lebanon; ^4^ Faculty of Medicine American University of Beirut Beirut Lebanon; ^5^ Doctoral School “Abbé Grégoire” National Conservatory of Arts and Crafts Paris France

**Keywords:** confidence, intravenous cannulation, medical education, task trainer‐based medical education

## Abstract

**Background and Aims:**

Peripheral intravenous (IV) cannulation is a fundamental clinical skill that requires both technical precision and effective patient communication. Despite its frequency in clinical practice, many medical trainees report insufficient confidence and limited opportunities for supervised practice. Simulation‐based medical education has emerged as a strategy to bridge this gap; however, its impact on both technical and nontechnical competencies warrants further evaluation. This study was done to assess the effect of a simulation‐based training module on medical students' technical skills (TS), nontechnical skills (NTS), and self‐reported confidence in peripheral IV cannulation.

**Methods:**

This single‐group pre–post interventional study included 74 3rd‐ and 4th‐year medical students. Participants underwent a structured task trainer‐based training session on IV cannulation. Performance was assessed before (T2) and after (T4) the intervention using a modified Objective Structured Assessment of Technical Skills (OSATS), evaluating both TS and NTS (maximum score: 16 each). Self‐reported confidence was measured on a 10‐point Likert scale. Paired t‐tests were used to compare pre and postintervention confidence, NT, and NTS scores.

**Results:**

Baseline confidence increased from 4.25 ± 1.22 to 7.32 ± 1.31, a mean increase of 3.07 points (*p* < 0.001). Mean NS scores increased from 4.4 ± 2.1 to 12.1 ± 1.3, a mean increase of 7.7 points (*p* < 0.001), while NTS scores increased from 6.9 ± 2.5 to 12.9 ± 2.1, a mean increase of 6.0 points (*p* < 0.001). Overall satisfaction was high, with a mean score of 4.4/5, and most participants reporting perceived usefulness in clinical practice.

**Conclusion:**

Task trainer‐based training significantly enhances medical students' technical performance, NTS, and confidence in peripheral IV cannulation within a controlled learning environment. These findings suggest that a structured case‐based simulation session may be associated with immediate improvements in procedural performance and self‐reported confidence. Controlled studies with longer‐term follow‐up are needed to determine the causal and sustained effects of this training approach.

**Abbreviations**
Human Ethics: informed consent was gained from each participant during study explanation and prior to starting
IV: intravenous
NTS: nontechnical skills
OSATS: Objective Structured Assessment of Technical Skills
SD: standard deviation
SRCL: self‐reported confidence level
T: time point
TS: technical skills

## 1 Introduction

Peripheral intravenous (IV) cannulation is one of the most frequently performed invasive procedures in clinical practice [1]. Although it is considered a basic clinical skill, successful cannulation requires coordination of patient assessment, equipment preparation, vein selection, aseptic technique, needle manipulation, and appropriate postprocedural management [2]. Difficulties with cannulation can result in repeated attempts, patient discomfort, delays in care, and potentially avoidable complications [1]. Early development of competence in this procedure is therefore important for medical students before they undertake greater responsibility in clinical settings [2, 3].

Simulation‐based education has become an important component of medical training, particularly for procedural skills that require repeated practice before clinical application [4]. Simulation allows learners to practice procedures in a controlled environment without exposing patients to unnecessary procedural attempts and can provide opportunities for standardized assessment of technical performance [5]. In addition to procedural execution, case‐based simulation can incorporate clinical assessment and decision‐making, allowing learners to integrate cognitive and psychomotor skills within a structured learning environment [6, 7].

Simulation‐based approaches have been used to facilitate procedural skills acquisition by providing learners with opportunities for repetitive practice and standardized assessment [6, 8, 9, 10]. Previous studies have reported improvements in procedural performance and learner confidence following simulation‐based training [11]. In skills training, Objective structured assessment of technical skill (OSATS)‐based assessment continues to be an effective method to structure objective evaluations of procedural performance [12, 13]. However, confidence and technical competence are related but distinct outcomes, and improvement in one does not necessarily establish competence in the other. Moreover, procedural training that incorporates a clinical case can provide an opportunity to assess nontechnical skills (NTS) and clinical decision‐making alongside technical performance [14].

Despite the widespread use of simulation and task training in medical education, opportunities for structured training in peripheral IV cannulation may vary among medical students because clinical exposure depends on patient availability, supervision, and procedural opportunities. A standardized educational intervention that combines a clinical case with hands‐on procedural practice may provide a practical approach to addressing this gap. Thus, this study aims to evaluate the efficacy of a task trainer‐based peripheral venous line placement module on the theoretical and technical skills of IV placement in medical students, while assessing their confidence performing this procedure.

## 2 Methods

### 2.1 Study Design and Population

This study used a single‐group pretest–posttest design to evaluate immediate changes in medical students' technical skills, NTS, and self‐reported confidence following a structured case‐based simulation training session. This design was selected because the educational intervention was delivered as a standardized training activity to the participating student cohort, with the primary objective of evaluating within‐participant changes following the intervention. A concurrent untreated comparator group was not included because the training was implemented as a structured educational activity for the participating cohort, and withholding the training from eligible participants solely for the purpose of creating a control group was not considered feasible within the educational setting. The pretest–posttest design allowed each participant to serve as their own reference, thereby reducing the influence of between‐participant differences when evaluating immediate changes following training. Because there was no concurrent control group, however, the observed pre–post changes cannot be attributed exclusively to the training intervention, and the study was designed primarily to evaluate immediate within‐participant change rather than establish definitive causal effectiveness [15].

The study comprised 74 individuals in total, of which 37 were males (50%), and 37 were females (50%). All participants received a brief introduction explaining the goals and methods of the study prior to taking part. Before being enrolled, subjects had to fill out an initial online questionnaire to provide their informed consent. Participants who had already attended a session on IV insertion or who declined to participate were not included.

The study comprised six task trainer sessions overall. Every session adhered to a standardized framework founded on fundamental medical simulation concepts. As shown in Figure 1, each session was split into five major steps in order to standardize the evaluation approach.

**Figure 1:**
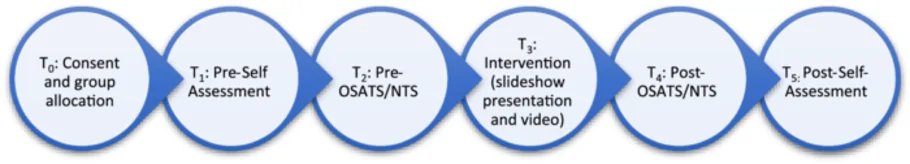
Overview of the workshop.

### 2.2 Data Collection and Procedures

Participants answered an anonymous questionnaire to assess the effectiveness of these clinical reasoning sessions. Two primary criteria were the focus of the assessment: (1) level of satisfaction and (2) perceived importance of the sessions.

#### 2.2.1 Technical Learning of Routine Procedures

The educational intervention consisted of a structured procedural skills session using a peripheral IV cannulation task trainer. Participants first received instruction and demonstration of the standardized cannulation technique and were subsequently provided an opportunity to practice the procedure on the task trainer. The session focused on procedural sequence, aseptic technique, equipment handling, vein selection, cannulation technique, and postcannulation management. Participants subsequently underwent postintervention assessment using the same procedural skills assessment framework. The journey from baseline assessment to posttraining evaluation was represented by the successive stages of the simulation‐based training, which were labeled T0 through T6. The intervention is described in accordance with published recommendations for reporting healthcare simulation research [16].

**T0:** Following completion of the online survey, participants were randomly divided into groups of 15 students, with one monitoring specialist and up to 3 senior residents or nurses monitoring the intervention and session, thus making a learner‐to‐faculty ratio of 1:3. They were then invited to participate in a 4‐h task trainer session at the university simulation center, which was led by senior anesthesia residents and experienced nurses. A brief overview of the many actions scheduled for that day was provided. To ensure their anonymity, the participants were then asked to choose at random a number that would serve as their ID for the duration of the session.

T1: A Google Forms‐based Pre‐Self‐Assessment survey was disseminated via QR code. The method of permission for taking part in our study and simulation session was the first question on the form. Gender, academic year, the approximate number of IV insertions they had previously attempted, and their self‐reported confidence level (SRCL) in completing IV insertions on a scale of 1 to 10 were among the sociodemographic questions that followed. The participants' fundamental understanding of IV insertion, including its indications, contraindications, and essential components, was assessed using six more questions.

T2: Participants were then asked to replicate a full IV insertion procedure on a mannequin with the assistance of a junior resident. Each participant was given a commercially available peripheral IV cannulation task trainer model consisting of a life‐sized adult arm with anatomically positioned superficial veins, typically including the cephalic, basilic, and dorsal hand veins. The veins were composed of palpable, compressible tubing connected to a closed fluid reservoir system containing artificial blood, allowing for realistic flashback upon successful cannulation. The overlying skin was made of puncture‐resistant synthetic material designed to mimic the texture and elasticity of human tissue and to permit repeated needle insertions without significant degradation. The simulator was mounted on a stable platform to replicate standard patient positioning and to facilitate ergonomic practice. The intervention used a task trainer to provide physical fidelity for peripheral IV cannulation, while the accompanying clinical case provided contextual fidelity for clinical assessment and decision‐making. The session was not designed as an immersive, high‐fidelity simulation.

Senior residents were concurrently monitoring and grading the student's performance using a modified OSATS‐based assessment rubric [12], which evaluated both technical skills (TS) and NTS. At the conclusion of the assessment sheet, a final “pass or fail” question was included as general feedback. Hands‐on practice continued until participants completed the standardized procedural sequence and the instructor determined that the session objectives had been addressed.

As indicated in Table 1, four domains were evaluated separately for both TS and NTS, each with a maximum score of four points, for a total potential score of sixteen points for each skill set.

**Table 1:** Rubric for assessment of TS and NTS at *T*_2_ and *T*_4_.

Domain	Category	Assessment criteria (1 point each)
TS Maximum Score: 16 points	Equipment gathering	Failed to request necessary equipment Requested only essentials (e.g., cannula without full setup) Requested antiseptic solution and tourniquet Requested all required materials, including IV cannula, gloves, antiseptic wipes, tourniquet, gauze, tape/dressing, flush syringe, and disposal bin
	Performance of IV insertion	Selected appropriate vein and site Applied tourniquet correctly Performed proper skin antisepsis and allowed drying time Inserted cannula using appropriate technique and angle
	Recognition of IV placement & complications	Confirmed correct placement (flush without resistance/swelling) Recognized signs of infiltration/extravasation Identified failed attempt and adjusted appropriately Maintained aseptic technique throughout
	Postprocedure management	Secured cannula properly with dressing Disposed of sharps safely Performed proper hand hygiene Documented procedure and site
NTSMaximum Score: 16 points	Explanation of procedure	Introduced self and verified patient identity Explained the purpose and steps of IV insertion Obtained informed consent Reassured the patient and addressed concerns
	Adequate positioning	Ensured privacy and appropriate exposure of the insertion site Positioned the patient comfortably with arm supported Selected appropriate limb positioning to optimize venous access Positioned self‐ergonomically for safe and effective insertion
	Reporting of findings	Used appropriate medical terminology Accurately reported success or difficulty of insertion Documented IV site, cannula size, and number of attempts Properly signed and dated the report
	Professional behavior and communication	Maintained professional and respectful demeanor Demonstrated empathy and patient‐centered attitude Communicated clearly and confidently throughout Thanked the patient and ensured privacy

T3: After the first performance, an experienced senior nurse extensively discussed vascular considerations, indications, contraindications, the step‐by‐step IV insertion procedure, and methods for reporting results during a 15‐min PowerPoint presentation. “Barbara Bates: A Guide to Physical Examination and History Taking, a Guide to Clinical Thinking, Twelfth Edition” [17] used as the basis for the lecture. A video from the same reference that showed the IV insertion steps was shown after the discussion.

T4: Under the identical circumstances as in T2, each participant was asked to repeat the IV insertion task trainer session on the mannequins. The same evaluators used the same evaluation method to test their TS and NTS a second time. Following the scenario and procedural assessment, participants participated in a structured debriefing facilitated by the instructor. The debriefing focused on clinical decision‐making, identification of errors, procedural performance, and areas for improvement. Instructors provided verbal feedback based on the participant's performance and assessment checklist. Each participant had the opportunity to repeat the procedure as often as needed; however, these repetitions were not included in this study and were solely for the improvement of learning for each student. The hands‐on component incorporated repeated practice with instructor feedback.

T5: Participants had access to a Post‐Self‐Assessment questionnaire by scanning a second QR code. Questions concerning their level of satisfaction regarding the task trainer session were also included.

### 2.3 Learning Objectives

The learning objectives were to enable participants to recognize indications and relevant clinical red flags in a patient requiring peripheral IV access, identify an appropriate initial management approach based on the clinical scenario, prepare the equipment required for peripheral IV cannulation, perform peripheral IV cannulation using an appropriate aseptic technique, demonstrate appropriate procedural communication and clinical decision‐making, and recognize and appropriately respond to common procedural or clinical problems encountered during IV cannulation.

### 2.4 Questionnaires

#### 2.4.1 Pre and Post Workshop Declarative Knowledge and Confidence

In order to assess declarative knowledge and SRCL in completing IV placement, participants filled out questionnaires both before and after the training. To guarantee measurement consistency, the same assessment scale was employed both before and after the intervention.

The Haute Autorité de Santé (HAS) framework served as the basis for the questionnaire's development, thus increasing compliance with accepted criteria for evaluating clinical competence.

#### 2.4.2 General Satisfaction Questionnaire

To assess participants' general satisfaction with the training program, a different survey was sent out. A 5‐point Likert scale, with 1 representing “strongly dissatisfied” and 5 representing “strongly satisfied,” was used to record responses.

The primary outcomes of interest were the changes in self‐reported confidence, technical skills, and NTS between the preintervention and postintervention assessments. Participant satisfaction and perceptions of the training were evaluated as descriptive secondary outcomes and were not included in the primary hypothesis‐testing analysis. No subgroup analyses were prespecified or performed.

### 2.5 Statistical Analysis

Continuous variables were summarized using means and standard deviations (SD), while categorical variables were summarized using frequencies and percentages. The primary analyses evaluated within‐participant changes in self‐reported confidence, technical skills, and NTS between the preintervention and postintervention assessments. Paired *t*‐tests were used to compare the corresponding pre and postintervention scores. All statistical tests were two‐sided, and A two‐sided *p* value of < 0.05 was prespecified as the threshold for statistical significance. Results are presented with the corresponding pre and postintervention means and SDs, mean changes, and *p* values. Satisfaction outcomes were summarized descriptively and were not subjected to additional hypothesis testing. No subgroup analyses were prespecified or performed. Statistical analyses were performed using IBM SPSS Statistics for Windows, version 28.0 (IBM Corp., Armonk, NY, USA). Statistical reporting followed recommendations for clinical research reporting and the Statistical Analyses and Methods in the Published Literature (SAMPL) guidelines [18, 19].

### 2.6 Ethical Considerations

This study was reviewed and approved by the Ethical Review Board of the Holy Spirit University of Kaslik (USEK), Jounieh, Lebanon (Approval number: EN‐CETH‐001). All participants were informed about the objectives of the study prior to enrollment. Written informed consent was obtained from all participants before participation. The study procedures were conducted in accordance with the ethical principles outlined in the Declaration of Helsinki.

## 3 Results

### 3.1 Demographics

The study included seventy‐four 3rd and 4th year medical students, of whom 37 (50%) were men, and 37 (50%) were female. Third‐year medical students comprised 42 (56.8%) of the participants, while fourth‐year medical students comprised 32 (43.2%).

### 3.2 Confidence Level, Technical and NTS

The majority of students 93.23% (69) did not feel sufficiently trained to insert a peripheral venous line before the task trainer session; Their SRCL for this procedure in T1 had an average of 4.25 ± 1.22 E.T. The SRCL increased significantly to 7.3 ± 1.31 after SBME (*p* < 0.001).

At baseline, the mean self‐reported confidence score for peripheral IV cannulation was 4.25 ± 1.22 on a 10‐point scale and increased to 7.32 ± 1.31 following the intervention, representing a mean increase of 3.07 points (*p* < 0.001). Mean technical skills scores increased from 4.4 ± 2.1 to 12.1 ± 1.3 out of 16, corresponding to a mean increase of 7.7 points (*p* < 0.001). Similarly, mean NTS scores increased from 6.9 ± 2.5 to 12.9 ± 2.1 out of 16, representing a mean increase of 6.0 points (*p* < 0.001). The pre and postintervention values and corresponding changes are summarized in Table 2.

**Table 2:** Pre and postintervention confidence, technical skills, and nontechnical skills scores.

Variable	Mean ± standard deviation		*p*‐value
Confidence scale to complete the NPV (out of 10):	Preintervention	4.25 ± 1.22	< 0.001
	Postintervention	7.32 ± 1.31	
	Mean change	+3.07	
Technical skills (score)/16	Preintervention	4.4 ± 2.1	< 0.001
	Postintervention	12.1 ± 1.3	
	Mean change	+7.7	
Nontechnical skills (score)/16	Preintervention	6.9 ± 2.5	< 0.001
	Postintervention	12.9 ± 2.1	
	Mean change	+6.0	

### 3.3 Satisfaction

As shown in Tables 3 and 4, at the end of this exercise, the average mark given by the students for the training was 4.4/5 (SD 0.4). Since none of the participants had ever taken part in a live or virtual IV insertion simulation session, none of them were disqualified from our study. In addition to practice, the majority of our sample 57 (77%) said that a live presentation in a simulation center would be the most effective simulation strategy.

**Table 3:** Summary of the final evaluation of the venous line exercise by students.

Domain	Answer	*n*	%
Satisfaction	Very well	68	91.8
	Ok	6	8.2
Quality of training	Very well	74	100
	Ok	0	0
Meeting the needs of trainees	Very well	73	98.6
	Ok	1	1.4
Usefulness of the skills acquired in daily practice	Yes	73	98.6
	No	1	1.4
Recommendation of the exercise to other trainees	Yes	73	98.6
	No	1	1.4

**Table 4:** Summary of the Likert scale satisfaction survey filled by the participants.

Variable (*N* = 74)	Mean ± SD
Overall score for this course (from 1 to 5):	4.4 (0.4)
How satisfied are you? (from 1 to 5):	4.1 (0.5)
To what extent did the presenter outline the objectives? (from 1 to 5):	4.1 (0.5)
What is your assessment of the duration of the training? (from 1 to 5):	3.6 (1.1)

## 4 Discussion

This study used a single‐group pretest–posttest design to evaluate immediate changes in procedural performance and self‐reported confidence following task trainer‐based training. The paired design allowed each participant to serve as their own reference when comparing preintervention and postintervention measurements. The analysis demonstrated improvements across all three assessed performance‐related outcomes. These findings provide evidence of immediate improvement following the training session, although the absence of a concurrent control group limits the ability to attribute the observed changes exclusively to the intervention.

Following the task trainer exercise, all three prespecified performance‐related outcomes demonstrated statistically significant improvement. The magnitude of change was also substantial: self‐reported confidence increased by 3.07 points on a 10‐point scale, while technical and nontechnical skill scores increased by 7.7 and 6.0 points, respectively, on 16‐point scales. These findings suggest that the intervention was associated with improvement across both procedural performance and self‐reported confidence. However, the magnitude of these changes should be interpreted within the context of the single‐group pre–post design and the immediate postintervention assessment.

One of the study's most relevant findings is the significant increase in confidence. Procedural proficiency requires confidence, particularly for novice trainees who may understand a procedure's theoretical processes but are hesitant to provide actual patient care [20]. The 3.07‐point increase in self‐reported confidence following the simulation session suggests that the intervention was well received as a controlled setting in which participants could practice and repeat the procedure without exposing patients to unnecessary procedural attempts. Although increased confidence may facilitate willingness to engage in future procedural learning opportunities, the present study did not assess whether the observed increase in confidence translated into greater participation or improved performance during actual clinical care [20].

The findings show that task training has a significant educational impact at the technical skill level. The substantial rise in technical scores indicates better performance of the procedural phases, which includes equipment preparation, adherence to aseptic technique, vein selection, catheter insertion, and methodical procedure completion [21, 22]. Although peripheral venous line insertion is frequently thought of as a simple technique, its execution requires coordination, sequencing, and repeated practice. The study's improvement adds credibility to the claim that simulation is especially useful for teaching invasive bedside procedures that call on procedural consistency and psychomotor control [23, 24, 25, 26]. The observed increase in technical skill scores, from 4.4 ± 2.1 to 12.1 ± 1.3 out of 16, represents a substantial improvement in performance on the study‐specific assessment following the training session.

The considerable improvement in NTS is an equally noteworthy finding. Although venous line insertion is frequently viewed only as a technical procedure, it actually calls for professionalism, communication, assurance, and a patient‐centered mindset [27]. Introducing yourself, outlining the process, gaining cooperation, reducing patient fear, and acting appropriately during the interaction are all requirements for students. After the simulation, participants' NTS ratings increased, suggesting that the exercise improved more than just the mechanical insertion steps. This is a crucial issue because, rather than considering technical performance, communication, and professional behavior as distinct skills, incorporating clinical context into simulation may enhance the relevance of procedural training by allowing learners to integrate technical performance with communication and professional behaviors.

The overall assessment of the training provided by the participants further supports the intervention's educational value. The training received an average score of 4.4 out of 5 from students, indicating a good level of satisfaction. Additionally, the final evaluation revealed very positive perceptions in a number of areas: 98.6% of participants said that the skills they had learned were beneficial in their daily practice and that they would suggest the exercise to other trainees, and the majority of participants rated the session highly in terms of satisfaction, training quality, and meeting trainees' needs. These findings imply that the session was well‐received by the students in addition to being successful in terms of performance. This is significant because whether or not such training can be stably included into studentship programs or skill curriculum depends largely on acceptance and perceived usefulness [28].

Simultaneously, the final evaluation also brought to light an important point: although overall satisfaction and score were good, the training length assessment was lower than the other evaluation domains, averaging 3.6 ± 1.1 out of 5. This might mean that some participants thought the program was too long, exhausting, or not well‐timed. This point is helpful for the model's future development. It implies that even while the session's structure and content were valued, the learning process may be improved by making changes to the timing, group dynamics, or ratio of lecture to practical application [29, 30]. High content satisfaction and lower length satisfaction in educational interventions may indicate the need for improved pacing rather than an issue with the training itself [31].

Particularly for students who are anticipated to execute peripheral venous line placement often during early clinical practice, this study provides significant evidence in support of simulation‐based training. The findings suggest that task trainer‐based training may improve immediate procedural performance and self‐reported confidence in a controlled learning environment. The concurrent improvement in technical and nontechnical skill scores indicates that the intervention addressed both procedural execution and communication‐related behaviors within the simulated setting. However, these findings should not be interpreted as evidence of improved clinical competence or preparedness for independent patient care, as clinical performance and patient outcomes were not assessed.

Several methodological considerations should be considered when interpreting these findings. First, the single‐group pre–post design does not permit definitive causal attribution of the observed improvements to the training intervention because no concurrent comparator group was included. The design was selected to evaluate immediate within‐participant changes following a standardized educational session, but a control group was not feasible within the educational setting in which the training was delivered. Second, several threats to internal validity are inherent to a single‐group pre–post design. These include history, whereby events or educational experiences occurring between the pre and postintervention assessments could contribute to changes in performance; maturation, reflecting changes in learners over the assessment interval; testing effects, whereby exposure to the baseline assessment may improve subsequent performance; and regression to the mean, particularly if baseline performance was relatively low. In addition, the immediate postintervention assessment may reflect short‐term familiarity with the task rather than durable acquisition of procedural performance. Because the study did not include a control group, these alternative explanations cannot be separated from the effect of the intervention. Future studies using randomized or controlled designs with delayed follow‐up assessments would provide stronger evidence regarding the causal and sustained effects of the training. Finally, if assessors were aware of whether a performance was evaluated before or after the intervention, observer expectations could have contributed to differences in postintervention ratings.

Therefore, future research might concentrate on assessing the long‐term retention of these improvements, contrasting students educated using simulation with those trained through traditional bedside exposure alone, and determining if repeated simulation sessions result in additional improvement. Evaluating how this kind of training affects real‐world clinical outcomes, such the success rate of initial attempts, procedural issues, or patient satisfaction during venous line insertion, might also be beneficial. The evidence would be strengthened, and the results' external validity would be enhanced by extending the intervention to bigger groups and several locations.

## 5 Conclusion

In this single‐group pre–post study, case‐based simulation training was associated with immediate improvements in technical skills, NTS, and self‐reported confidence in peripheral IV cannulation within the simulated environment. These findings support the potential utility of structured simulation for procedural skills training; however, they do not establish improvement in clinical competence, retention of skills, or patient outcomes. Controlled studies with longer‐term follow‐up and assessment in clinical settings are needed to determine whether improvements demonstrated in simulation translate into clinical performance.

## Author Contributions

**Maher Abdessater:** conceptualization, writing – original draft, writing – review and editing, methodology, project administration, supervision. **Ramy Touma Sawaya:** writing – original draft, writing – review and editing, formal analysis, data curation. **Sandra Elias:** conceptualization, writing – original draft, writing – review and editing, supervision, project administration. **Joanne El Turk:** writing – original draft, writing – review and editing, methodology, formal analysis. **Pascal Roquet:** supervision, writing – original draft, writing – review and editing. **Raghid El Khoury:** supervision, writing – review and editing, writing – original draft.

## Funding

This research received no specific grant from any funding agency in the public, commercial, or not‐for‐profit sectors. As no external funding was received, there was no funding source involved in the study design; collection, analysis, or interpretation of data; writing of the report; or the decision to submit the manuscript for publication. All authors have read and approved the final version of the manuscript. Maher Abdessater had full access to all of the data in this study and takes complete responsibility for the integrity of the data and the accuracy of the data analysis. Maher Abdessater affirms that this manuscript is an honest, accurate, and transparent account of the study being reported; that no important aspects of the study have been omitted; and that any discrepancies from the study as planned (and, if relevant, registered) have been explained.

## Consent

IRB approval was obtained from the Ethical committee in the Holy Spirit University of Kaslik, and all experiments were performed in accordance with the Declaration of Helsinki and other national and studentationally relevant guidelines and regulations.

## Conflicts of Interest

The authors declare no conflicts of interest.

## Data Availability

Data sharing not applicable to this article as no datasets were generated or analyzed during the current study. The authors confirm that all data supporting the findings of this study are available within the article and its tables.
